# User Acceptance of Wrist-Worn Activity Trackers Among Community-Dwelling Older Adults: Mixed Method Study

**DOI:** 10.2196/mhealth.8211

**Published:** 2017-11-15

**Authors:** Arjun Puri, Ben Kim, Olivier Nguyen, Paul Stolee, James Tung, Joon Lee

**Affiliations:** ^1^ Health Data Science Lab School of Public Health and Health Systems University of Waterloo Waterloo, ON Canada; ^2^ Department of Electrical and Computer Engineering University of Waterloo Waterloo, ON Canada; ^3^ School of Public Health and Health Systems University of Waterloo Waterloo, ON Canada; ^4^ Department of Mechanical and Mechatronics Engineering University of Waterloo Waterloo, ON Canada

**Keywords:** health, mHealth, fitness trackers, older adults

## Abstract

**Background:**

Wearable activity trackers are newly emerging technologies with the anticipation for successfully supporting aging-in-place. Consumer-grade wearable activity trackers are increasingly ubiquitous in the market, but the attitudes toward, as well as acceptance and voluntary use of, these trackers in older population are poorly understood.

**Objective:**

The aim of this study was to assess acceptance and usage of wearable activity trackers in Canadian community-dwelling older adults, using the potentially influential factors as identified in literature and technology acceptance model.

**Methods:**

A mixed methods design was used. A total of 20 older adults aged 55 years and older were recruited from Southwestern Ontario. Participants used 2 different wearable activity trackers (Xiaomi Mi Band and Microsoft Band) separately for each segment in the crossover design study for 21 days (ie, 42 days total). A questionnaire was developed to capture acceptance and experience at the end of each segment, representing 2 different devices. Semistructured interviews were conducted with 4 participants, and a content analysis was performed.

**Results:**

Participants ranged in age from 55 years to 84 years (mean age: 64 years). The Mi Band gained higher levels of acceptance (16/20, 80%) compared with the Microsoft Band (10/20, 50%). The equipment characteristics dimension scored significantly higher for the Mi Band (*P*<.05). The amount a participant was willing to pay for the device was highly associated with technology acceptance (*P*<.05). Multivariate logistic regression with 3 covariates resulted in an area under the curve of 0.79. Content analysis resulted in the formation of the following main themes: (1) smartphones as facilitators of wearable activity trackers; (2) privacy is less of a concern for wearable activity trackers, (3) value proposition: self-awareness and motivation; (4) subjective norm, social support, and sense of independence; and (5) equipment characteristics matter: display, battery, comfort, and aesthetics.

**Conclusions:**

Older adults were mostly accepting of wearable activity trackers, and they had a clear understanding of its value for their lives. Wearable activity trackers were uniquely considered more personal than other types of technologies, thereby the equipment characteristics including comfort, aesthetics, and price had a significant impact on the acceptance. Results indicated that privacy was less of concern for older adults, but it may have stemmed from a lack of understanding of the privacy risks and implications. These findings add to emerging research that investigates acceptance and factors that may influence acceptance of wearable activity trackers among older adults.

## Introduction

### Smart Wearable Devices and Older Adults

Today, Canadian older adults are leading longer, healthier, and more active lives compared with older adults from previous decades [1-3]. Successful aging is achieved through aging-in-place, a concept that depicts the continued living at home and to do so while maintaining independence, social contact, and dignity [4-6]. Aging-in-place has profound health and mental benefits [7-9]; is more cost-effective than institutionalized care [7,8,10]; and is perceived as more desirable, graceful, and fulfilling among the aging cohort [4,11].

With the increased desirability to age in place, numerous technologies have emerged with the aim of supporting aging-in-place with diverse purposes, including enhancing safety through providing medication reminders [12,13], improving social interactivity through video telephony [12,14,15], and maintaining capacity to carry out daily activities functions via electronic memory aids [16,17]. In recent years, off-the-shelf smart wearable devices such as heart rate monitors and physical activity trackers have seen tremendous growth [18]. Consumer research indicates that baby boomers are the next primary users of smart wearable technology as they are the fastest growing wearable activity tracker users [18,19]. More importantly, patients and providers equally anticipate a greater role of wearable activity trackers in managing health and achieving high quality of care and patient satisfaction [20]. The rapid growth of wearable activity trackers may have originated from the opportunity it provides for aging-in-place as a tool that can enable self-management of chronic diseases, remote monitoring by clinicians, and collecting clinically relevant data that can fuel big data analytics [21,22]. To seize this opportunity, it is critical to understand the factors and processes involved in adopting and using smart wearable activity trackers among older adults. Understanding the acceptance and use of smart wearable activity trackers are especially important for enabling aging-in-place, as ongoing and voluntary use is critical to accurate and comprehensive data collection for technology-driven interventions [23]. However, there currently exists little research that appropriately and adequately explores older adults’ attitudes toward, as well as acceptance and usage of, smart wearable activity trackers [24,25].

### Literature Review on Wearable Acceptance

Research activities for smart wrist activity trackers tend to focus on younger populations [26,27]. An increase in gerontological research has been observed because of the increased adoption of smart wearable activity trackers among older adults and the recognition of their greater potential health benefits to older adults [28-30]. Previous research studies identified that older adults in general perceived smart wearable activity trackers as easy to use, useful, comfortable, and acceptable in the short term [28] and long term [29]. These 2 studies used wearable activity trackers that clip onto the belt or pocket, which can affect its acceptance differently than wearable activity trackers that are worn on wrists like a bracelet. One study examined the acceptance of both wearable activity trackers that are worn on wrists and clip-ons and traditional pedometers by older adults [30]. This study reported higher acceptance of wearable activity trackers, either clip-on or wrist worn, over pedometers among older adults [30]. Furthermore, older adults expressed that wrist-worn activity trackers are preferred over clip-ons because of a less likelihood of losing or breaking them [30].

The first impression is often determined by the style, but a long-term adoption of wearable activity trackers is often influenced by an array of factors such as comfort and usability [31]. The short study duration of the previous smart wearable device research study left the investigation of the technology acceptance deficient and limited to the first impression [30]. The duration of the study ranged from 3 days to 7 days for participants [30]. Short-term technology acceptance may not be indicative of long-term acceptance as research indicates that use of smart wearables such as activity trackers tends to drop after the first few weeks of ownership [18]. This rapid drop of adherence to wearable activity trackers is also apparent in a younger population where more than 50% stopped using the device after 14 days and 75% at around 30 days [32]. Therefore, there is a need for a study with a longer duration than 7 days.

Existing research studies [28-30] examined the acceptance level solely based on the technology acceptance model (TAM) [33]. The theoretical constructs from TAM, including perceived usefulness, perceived ease of use, and other external variables such as comfort, were highly associated with older adults’ acceptance [28-30]. However, investigation of technology acceptance with TAM leaves out other critical factors that are important. TAM received an update (ie, TAM2) to put greater emphasis on technology acceptance within organizational settings consisting of additional theoretical constructs that describe the perceived usefulness that incorporates social influences, output quality, and result demonstrability [34]. Further iteration of update resulted in TAM3, which refined and added more theoretical constructs related to the perceived ease of use [35]. Another popular theoretical model for technology acceptance, the unified theory of acceptance and use of technology (UTAUT), which combined multiple technology acceptance and behavior change theories, also emphasizes the theoretical constructs related to social influences as well as individual characteristics such as age and gender as a moderator to behavior intention [36]. These newer theoretical constructs are missing in the TAM that the existing research studies used, yet the significant increase in these models’ performance in explaining technology acceptance is attributed to these newly added constructs [34-36].

These theoretical models aim to provide general determinants of technology acceptance. Subsequently, they lack context specificity and artifact specificity, but they can provide more relevant key predictors for technology acceptance [35]. There has been development of technology acceptance theoretical models that are specific to older adults such as social agent technologies [37] and Internet uses [38] or for emerging technologies such as wearable devices [39,40]. Gao et al [40] identified privacy as an important barrier for older adults to accept wearable technology. Equipment characteristics such as battery longevity, ergonomics, and aesthetics have also been identified as important factors for older adults [30,41]. Cost was found to be a significant factor in a systematic review that examined technologies for aging-in-place [17]. A similar pattern was noted in a previous wearable activity tracker study in which participants considered cost as one of the major barriers for future purchase [30]. This raises the question of sensitivity toward the cost of equipment as the tested devices’ prices ranged from US $60 to US $150, which is often considered low cost in the eyes of researchers [28]. To the best of our knowledge, no studies have examined the factors for acceptance of wrist-worn activity trackers among older adults using potential determinants outside TAM.

### Research Objective

The objective of this study was to assess acceptance and usage of wearable activity trackers in Canadian community-dwelling older adults in a free-living environment. This study extends the current literature by investigating additional potentially influential factors to TAM, unique to emerging technologies and older adults, including privacy concerns, facilitating conditions, perceived risks, subjective norm, and equipment characteristics.

## Methods

### Study Design

A sequential explanatory mixed method design was used to explore the study objective. The quantitative data were collected in the first phase of the study through a questionnaire that was developed specifically for this study. The questionnaire results were analyzed and a semistructured interview guideline was developed. In the second phase, semistructured interviews were conducted to further probe older adults’ experience with wrist-worn activity trackers and complement the quantitative analyses.

Research ethics approval for this study was obtained from the University of Waterloo Office of Research Ethics. All participants gave written informed consent.

### Procedures

#### Phase 1

This was a 6-week-long phase focused on quantitatively assessing older adults’ technology acceptance through the questionnaire. This phase applied a crossover study design in which participants were randomly assigned to 2 groups, one for the Microsoft Band and the other for the Xiaomi Mi Band. The crossover between the 2 groups happened at the end of the third week. In other words, each participant tried both wrist bands, for 3 weeks each, but the order of the devices was randomized. The questionnaire was administered twice to capture technology acceptance toward each device, at the end of the third and sixth week. Demographical information and information regarding previous experience with technology and wearable devices were also collected.

#### Phase 2

Semistructured interviews were conducted at the participant’s own residence in a private setting. A reflexive interview process, which allows for modification of the interview technique and content as needed, was adopted to overcome the researcher’s own postulations or presumptions of wearable activity trackers [42,43]. The first interview was reviewed to note early instances of potential biases in the form of leading questions, which was readjusted and corrected in the remaining 3 interviews.

### Recruitment

A convenience sampling technique was used to recruit 20 older adults aged 55 years and older from the cities of Kitchener, Waterloo, Cambridge, and Guelph in Ontario, Canada. Flyers were posted in at local community centers and recreational facilities with approval. Interested participants were instructed to contact the researcher via phone or email. Recruitment started in March 2016 and lasted for 2 months. Participants must have been able to wear 2 wearable activity trackers for 21 days each.

A purposive and criterion sampling was used to recruit 4 participants for the subsequent semistructured interviews from the 20 participants. The criteria for purposive sampling were determined by age and the degree of wearable activity tracker acceptance (ie, high acceptance, medium acceptance, and low acceptance).

### Equipment

#### Wearable Activity Trackers

**Figure 1:**
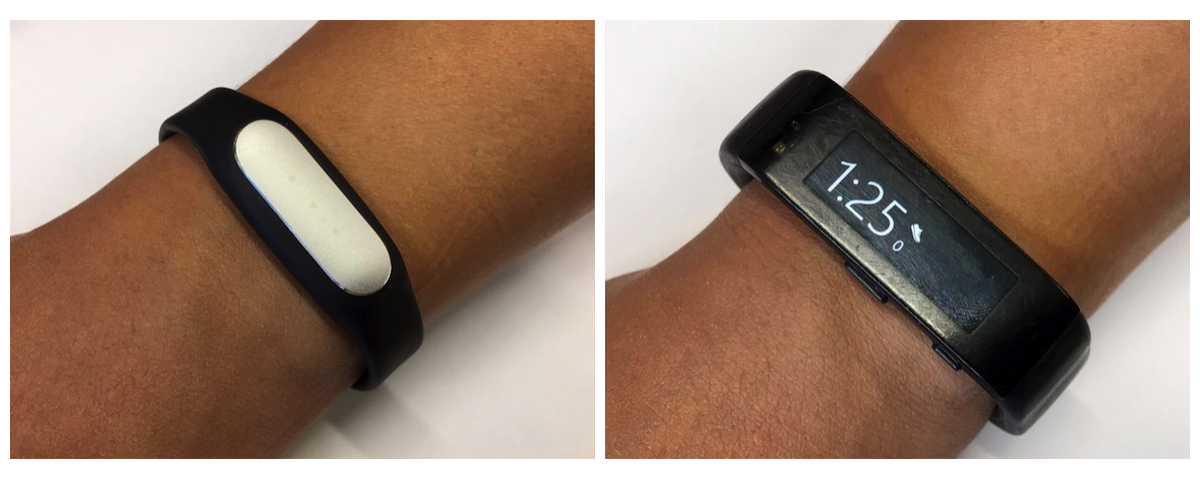
The Xiaomi Mi Band (left) and Microsoft Band (right).

Two consumer-level wearable activity trackers, the Microsoft Band and Mi Band (Figure 1), were selected based on their features (accelerometer, gyroscope, display), functionalities (step count, heart rate monitoring, recommendations), ergonomics (size and flexibility), and price. These 2 wearable activity trackers have immense differences in features, sensors, and price. As a result, these 2 devices represent the far ends of the consumer market spectrum, and the selection of devices was based on these differences.

The Microsoft Band offers an extensive sensor array and a touch screen liquid-crystal display (LCD; Table 1). The manufacturer’s quoted battery life is approximately 48 hours but varies based on individual usage. It is considerably more expensive than basic activity and heart rate trackers available at a cost of US $299 at launch.

The Mi Band offers a basic triaxial accelerometer and an optical heart rate sensor at a cost of US $20 at launch (Table 1). The Mi Band has an estimated battery life of almost 30 days, with real-world tests performance ranging from 45 days to 50 days. The Mi Band has 3 individual light-emitting diodes (LEDs) that provide activity progress by lighting up 1, 2, or all 3. The device is lightweight and can be worn on either the wrist or neck as a pendant, offering versatile placement. The placement of the Mi Band was limited to the wrist for this study.

**Table 1:** Two wearable activity trackers’ characteristics.

Characteristic	Mi Band	Microsoft Band
Display	LED^a^ dots	Touch LCD^b^ display
Battery life	30 days	48 hours
Cost	US $20	US $299
Sensors	Accelerometer, optical heart rate sensor	Accelerometer, optical heart rate sensor, gyroscope, galvanic skin response sensor, global positioning system, UV^c^ sensor, microphone

^a^LED: light-emitting diode.

^b^LCD: liquid-crystal display.

^c^UV: ultraviolet.

**Table 2:** Description of dimensions related to technology acceptance.

Dimensions	Description
Perceived usefulness	Perceived usefulness refers to improvements in one’s job performance but in the context of this study; it was adapted to refer to the degree to which using a technology can help monitor older adults’ health and support aging-in-place.
Perceived ease of use	Perceived ease of use has been established as a key indicator for user acceptance and is defined as “the degree to which a person believes that using a technology will be free from effort” [45].
Subjective norm	Subjective norm is another dimension that is representative of user acceptance, and it is defined as the likelihood of recommending the use of the said technology to individuals who are influential in the lives of the technology user [45] **.**
Facilitating conditions	A review of literature revealed varying classifications of facilitating conditions [38]. Facilitating factors are factors that can increase or decrease the effort required to use a technology such as availability, affordability, availability of training resources, and so on [46].
Privacy concerns	Privacy concerns is a novel dimension in the framework and has been included because of the emergent tendency of smart device and technology manufacturers to use Internet communication protocols to store and analyze data in the cloud, rather than on the particular device.
Perceived risks	Perceived risks have been established to be influential to consumer behavior and important when evaluating user acceptance of technology [47].
Equipment characteristics	Finally, equipment characteristics that can influence the technology acceptance were deemed an important dimension and described as one of the major factors in another study [41].

#### Smartphone

Both wearable activity trackers offer companion smartphone apps, and all participants were provided with an accompanying smartphone, the Motorola Moto E, with the app preinstalled and set up for use. The collected data from wearable activity trackers were transferred to the smartphones. The apps displayed the progress, patterns, and summaries. Participants were trained on how to use the wearable activity trackers and smartphone apps, but smartphone use was not mandatory.

### Data Collection and Analysis

#### Technology Acceptance Questionnaire

A 31-item, 5-point Likert scale, and an additional 6 multiple-choice items, self-reported, paper-based questionnaire for older adults was developed based on the fundamental dimensions that influence user acceptance of technology from TAM [33] and the sensor acceptance model [44] (Multimedia Appendix 1). The investigated key dimensions for wearable activity tracker acceptance were perceived usefulness, perceived ease of use, privacy concerns, perceived risks, facilitating conditions, subjective norm, and equipment characteristics (Table 2). Detailed description of the technology acceptance questionnaire, including each dimension and corresponding questions, is summarized in Multimedia Appendix 1.

#### Semistructured Interview

A crossover approach to data collection through the questionnaire captured the technology acceptance and finer granular information about various factors that may influence the acceptance. However, this design of study does not fully explain the contextual factors related to technology acceptance and the participants’ cognitive rationale behind the questionnaire results. To overcome this shortcoming, one-to-one semistructured interviews were conducted in the second phase [48]. A semistructured interview guide was developed to gain deeper insight into each dimension of technology acceptance.

### Analyses

#### Phase 1

The demographic information, previous technology use, and characteristics related to smart wearable devices were analyzed through descriptive statistics. For all quantitative analyses, cases with missing data were excluded, which occurred because some participants refused to answer some of the questions.

The acceptance of the wearable activity tracker was measured by the question L33: *Would you use the device you used during the last 21 days to continue to monitor or track your physical activity or health?* In a univariate analysis, several statistical tests were used to analyze participants’ responses to each Likert-scale question, with respect to user acceptance. First, the Student independent *t* test was performed to investigate the differences in the total mean score for the technology acceptance questionnaire between the Microsoft and Mi Bands. Second, the Wilcoxon signed-rank test was conducted to test for the differences in the dimensions in the technology acceptance questionnaire between the 2 devices. Third, Spearman rho was calculated to test whether a correlation between participants’ responses to the Likert-scale questions and user acceptance exists. Finally, Spearman rho was used to assess the relationship between the order in which the devices were provided and the acceptance so as to ensure that the order did not influence the acceptance.

In a multivariable regression analysis, the association between the 7 dimensions from the technology acceptance questionnaire (Table 2) and user acceptance was assessed using logistic regression. Each dimension was represented by the sum of the responses to the questions that belong to that dimension. Because there were fewer than 40 questionnaire responses after excluding missing data (each of the 20 participants completed 2 questionnaires for the 2 wrist bands), only 3 of the 7 dimensions were selected using backward stepwise feature selection and were included as covariates in the logistic regression model, so that the number of covariates did not exceed one-tenth of the number of cases. Using the 3 selected dimensions, leave-one-out cross-validation was performed to evaluate the logistic regression model’s user acceptance classification performance. As the performance metric, the area under the receiver operating characteristic curve (AUC) was calculated. Statistical significance was set at alpha=.05 for all statistical results.

#### Phase 2

Semistructured interviews were coded and themed using a directed content analysis strategy whereby the themes explored follow structure determined by concepts reviewed in literature, while also allowing for the discovery of the previously undiscovered or unmentioned data and themes [43]. All interviews were transcribed, and 2 researchers independently read the transcripts to familiarize themselves with the data. Predetermined codes based on the concepts and variables discovered during literature review were used. New data that were not represented by preexisting categories were then identified and analyzed. Codes were reviewed and combined into themes that appropriately and accurately described the interview data. This process was iterative as the researchers’ knowledge of the interview data increased. The themes generated by the 2 researchers were compared with to note similarities and differences and transformed into the overarching themes.

## Results

### Participant Characteristics

All 20 participants who enrolled completed the study. The respondents ranged in age from 55 years to 84 years (mean 64 years), and 60% (12/20) were female. Of the total, 18 participants (90%) used a computer on a daily basis and 14 (70%) personally owned a smartphone. Seventeen participants (85%) had heard of smart wearable devices, indicating a high degree of awareness among the group, though only one used a store-bought wearable activity tracker to monitor their health (Table 3).

**Table 3:** Participant characteristics and previous technology experience.

Characteristic		Value
Age in years, mean (range)		64 (55-84)
**Sex, n (%)**		
	Male	8 (40%)
	Female	12 (60%)
**Marital status, n (%)**		
	Married	15 (75%)
	Divorced	2 (10%)
	Separated	1 (5%)
	Widowed	2 (10%)
	Never married	0 (0%)
**Education level, n (%)**		
	High school	3 (15%)
	Some postsecondary	3 (15%)
	Completed postsecondary	6 (30%)
	Some postgraduate	1 (5%)
	Completed postgraduate	7 (35%)
**Income in CAD dollars**^a^**, n (%)**		
	Less than $20,000	2 (10%)
	$20,000-$39,999	5 (25%)
	$40,000-$69,999	3 (15%)
	$70,000-$99,999	7 (35%)
	$100,000-$149,999	2 (10%)
	$150,000 or more	0 (0%)
**Computer use, n (%)**		
	None	1 (5%)
	Once a month	0 (0%)
	Once or twice a week	1 (5%)
	Daily	18 (90%)
**Smartphone ownership, n (%)**		
	Own smartphone	14 (70%)
	Do not own smartphone	6 (30%)
**Smart wearable device ownership, n (%)**		
	Own smart wearable device	1 (5%)
	Do not own smart wearable device	19 (95%)
**Heard of smart wearable devices, n (%)**		
	Yes	17 (85%)
	No	3 (15%)

^a^Missing n=1.

**Table 4:** Acceptance per device.

Acceptance	Mi Band, n (%)	Microsoft Band, n (%)	Combined, n (%)
Yes	16 (80)	10 (50)	26 (65)
No	4 (20)	10 (50)	14 (35)

**Table 5:** Differences in the technology acceptance questionnaire scores per dimension for each device.

Dimensions (maximum score)	Mi Band score, mean (SD)	Microsoft Band score, mean (SD)	*P* value
Equipment characteristics (30)	29.05 (3.27)	22.35 (4.20)	<.001
Perceived ease of use (35)	25.45 (4.70)	27.55 (3.24)	.11
Facilitating conditions (10)	12.1 (1.59)	11.55 (1.50)	.12
Perceived usefulness (25)	18.3 (2.72)	17.25 (2.69)	.13
Privacy concerns (15)	11.1 (2.71)	11.55 (2.67)	.18
Perceived risks (15)	4.75 (1.29)	4.9 (1.55)	.72
Subjective norm (15)	11.10 (2.00)	11.35 (2.21)	.94

### Wearable Activity Tracker Acceptance

Overall, the wearable activity trackers had a moderate level of acceptance (26/40, 65%) among the community-dwelling older adults. The Mi Band had higher acceptance rate (16/20, 80%) than the Microsoft Band (10/20, 50%) (Table 4). The order in which participants received the devices was not correlated with the acceptance for the Mi Band and Microsoft Band (*P* value of .67 and .73, respectively).

### Technology Acceptance Questionnaire

Overall, participants’ experiences were similar between the Mi and Microsoft Bands when the individual dimensions were compared (Table 5). The participants’ total score to the technology acceptance questionnaire were higher for the Mi Band (72.16) than Microsoft Band (68.71), but the Student *t* test was statistically not significant (*P*=.16). The Wilcoxon signed-rank test revealed that the Mi Band scored significantly higher in the equipment characteristics dimension (*P*<.001; Table 5). Statistical significance was not found for the perceived ease of use dimension between the devices (*P*=.11), despite the Microsoft Band scoring significantly higher in 3 questions that measured perceived ease of use (*P*<.05; Multimedia Appendix 2). No other dimensions were significantly different between the devices.

### Dimensions Associated With Technology Acceptance

According to Spearman rho, technology acceptance was moderately correlated with 8 questions with correlation coefficients ranging from .31 to .49 (Table 6). Technology acceptance was most highly correlated with question L35, which asked participants about the price they were willing to pay for wearable activity trackers. Privacy concerns (L19 and L20) were negatively correlated in moderate strengths to technology acceptance.

**Table 6:** Correlation between technology acceptance questionnaire and technology acceptance.

Question item #	Corresponding dimension	Correlation coefficient (rho)	*P* value
L35: How much would you be willing to pay for the device you wore during the last 21 days?	N/A^a^	.49	.001
L19: I had no concerns about my privacy while wearing the device.	Privacy concerns	−.43	.006
L21: I have the knowledge necessary to use the device.	Facilitating conditions	.43	.006
L20: I am comfortable with my health data being shared with equipment manufacturers as long as it is shared anonymously.	Privacy concerns	−.38	.02
L13: I find the device easy to use.	Perceived ease of use	.35	.03
L12: The device’s smartphone application was easy to use.	Equipment characteristics	.34	.03
L2: I was afraid that the device would discover a major health issue.	Perceived risks	.32	.04
L18: I was able to put the device on in a reasonable amount of time.	Perceived ease of use	−.31	.05

^a^N/A: not applicable

**Table 7:** Multivariate logistic regression model with 3 selected features.

Dimensions	Odds ratio (95% CI)	*P* value
Facilitating conditions	2.51 (1.20-5.27)	.02
Privacy concerns	0.64 (0.36-1.13)	.12
Perceived risks	1.82 (0.85-3.90)	.13

**Table 8:** Semistructured participant characteristics.

Participant name	Age in years	Gender	Band acceptance		Current smart device ownership
			Mi Band	Microsoft Band	
Anita	65	Female	No	No	Blackberry
Paula	84	Female	Yes	No	None
Francine	65	Female	Yes	No	iPad
Greg	83	Male	No	No	Yes^a^

^a^Exact device model was not identified.

### Predictors of Wearable Activity Tracker Acceptance

Feature selection resulted in the 3 dimensions (facilitating conditions, privacy concerns, and perceived risks) to be used as covariates in the multivariate logistic regression model. The logistic regression results are shown in Table 7. AUC for this model was 0.79.

### Directed Content Analysis

#### Participant Characteristics

The 4 participants recruited for the semistructured interviews and their acceptance of each wearable activity tracker are described in Table 8. Unfortunately, we could not recruit a participant who accepted both wearable activity trackers to satisfy the criterion sampling. Pseudonyms were assigned to protect confidentiality. The directed content analysis resulted in 5 overarching themes.

#### Theme 1: Smartphones as Facilitators of Wearable Activity Trackers

The acceptance of a wearable activity tracker could be affected as the wearable activity trackers relied heavily on smartphones for visualizing the data. When participants were asked about their experience and attitudes related to smartphones, they reverberated perceived ease of use as an important factor for future intention to use. Participants voiced their concerns with regard to perceived ease of use through fear of forgetting to use, losing, and breaking smartphones, as well as the inconvenience of carrying them, which influenced their future intention to use. Participants stated:

To tell you the truth, I was afraid to use it just in case I broke it because I didn’t know anything about it.

Paula

I’m not one to take a phone with me.

Francine

Lack of experience with smartphones among participants led to two different outcomes in older adults’ desire to use smartphones in the future. First, Anita and Francine, who had been exposed to smartphone previous to the study, responded positively toward their experience of using smartphones. Anita noted that she liked “the convenience” of a smartphone and “the fact that you just swipe it...I like that.” Francine appreciated the immediacy and convenience with which smartphones can provide information:

I like the idea that the information is right there when you want it...I like the idea that it’s a source of information that is easily accessible…

Francine

They expressed their future intention to use smartphones and willingness to learn as follows:

Well, I have tried to use it more. So I guess it helped to—made it decide that maybe we need to—I need to work more on it and try and figure out what exactly I can do with it.

Anita

I would think that they are the way of the future.

Francine

On the other hand, Greg and Paula described their prior experience with smartphone as negative and casted their pessimistic attitudes toward future intentions as follows:

We are so far behind in the e-world that trying to cope with those type of things, out of our ignorance, is really sort of impossible at times.

Greg

I don’t know if I’m ready to have a smartphone and get rid of my house phone.

Paula

Extending the negative perceptions toward smartphones, they speculated the harmful effect of smartphones by describing them as a “distraction,” “deterrent to conversation,” and “enslaving of our young people.”

#### Theme 2: Privacy Is Less of a Concern for Wearable Activity Trackers

Participants perceived the wearable activity tracker data such as step counts, sleep hours and efficiency, physical activity level, and heart rate as not private and were open to sharing them. Additionally, there was an overall diminished sense of privacy due to widespread data sharing in other aspects of their lives, as shown below:

I mean privacy—I would not like somebody to be able to go into my bank account or into personal details like that. But privacy; how I live or what I do, that’s not a—not bothering me. No.

Paula

It's like the information you have about your salary, how much you pay for your house, how much you pay for rent, how much you paid for your car, whether you had sex last night or anticipate having it next year [laughs] you know, all that stuff…I can share anything in my life and I don’t get the feeling that I shouldn’t be sharing that with somebody.

Greg

Francine displayed a high level of trust in the system and compared it with her trust in the security of electronic medical records as follows:

So, if it's connected with my name, great. I mean there's all that information in my doctor's computer, which is linked to the hospitals and various other places I'm sure. Can't see why it can't be there.

Francine

Participants’ perception of wearable activity tracker data differs from traditional notions of private data. Participants had minimal privacy concerns that were specific to wearable activity trackers and believed that they would have no bearing on acceptance and future use.

### Theme 3: Value Proposition—Self-Awareness and Motivation

Participants perceived the information from wearable activity trackers useful for understanding the level and intensity of physical activity they are getting, as echoed by the quotes below:

I’d like to know how inactive I am. I’d also like to know how much sleep I really do get.

Francine

But it has helped in that I know when I do certain walks, approximately how many steps and when I do my classes, how many steps. Then I can get a rough idea but it’s the competitiveness in me I guess to.

Anita

The increased self-awareness motivated lifestyle changes. Anita attested to her increased motivation to increase physical activity level, whereas Paula used the wearable activity tracker to recognize when to slow down:

To make sure that I’m doing my 8000 steps a day or whether the number so I’m getting enough exercise…But I would still I think be more likely to keep up my step count if I was wearing it.

Anita

Moving and that kind of stuff, so that's made me realized yeah you should stop and you should settle down and take it easy.

Paula

Wearable activity trackers triggered them to educate themselves on health topics. Greg stated:

The only thing I was surprised about, I know about REM sleep, I've done a lot of reading on it. I learned from this that out of 5½ or 6 hours sleep, or what I thought was sleep, an hour and 20 minutes was in REM.

Greg

Not all information was valuable, available, or presented in the right format. Information lost its value when participants already understood their current health status. Furthermore, wearable activity trackers did not present resting heart rate in an easy-to-understand manner, which diminished its value. Greg said:

…interesting, but not necessarily useful…But I wasn’t worried about whether I was doing the right thing or not…I never did learn anything about my resting heart rate when I was sleeping.

Greg

### Theme 4: Subjective Norm, Social Support, and Sense of Independence

Anita and Francine expected that their friends and family would support their decision to use wearable activity trackers and did not foresee reasons for discouragement from them. Francine had revealed that she received support from her husband with the use of wearable activity trackers, demonstrating the availability of immediate social support. However, Greg and Paula indicated possibly disheartening opinions from friends and family, followed by positive outlooks:

...my youngest daughter might think it is stupid…but I don’t think my oldest daughter would have anything against it.

Paula

The concept of subjective norm was closely tied to the sense of independence. This was implicitly dissolved in answers that reassured that it was ultimately their decision to use:

…at first, they [friends and family] might think that I’m a, what do you call the, hypochondriac. But I don’t care. And they will eventually come around to seeing that I’m taking it as an adult self-interest, a self-directive interest in my own being, my wellbeing.

Greg

That’s my opinion, if I want to do it, that’s up to me to do it. If I want to walk…what I want to do, I do.

Paula

The significance of the sense of independence to participants surfaced more explicitly when they were asked how much social support their family and friends can provide. Greg stated:

I’m sure it’s there [the support] but it means taking their time, and making my problem their problem. And that’s hard for me to do because of my own attitudes about independence I think. I really resent supervision, which is intrusive and demanding; kinds of stuff like that within the family.

Greg

The importance of older adults’ independence was highlighted and was demonstrative of an impediment in reaching out for loved one’s support in using wearable activity trackers. As a result of this hesitance, older adults may face reduced social approval and support, which may potentially affect the acceptance and future use of wearable activity trackers.

### Theme 5: Equipment Characteristics Matter—Display, Battery life, Comfort, and Aesthetics

The equipment characteristics of the 2 wearable activity trackers varied significantly. Participants confirmed that display, battery life, aesthetics, and comfort had a significant influence on device acceptance.

All participants approved of the significant value of LCD display on the Microsoft Band for quickly accessing information compared with the Mi Band. The Mi Band relied on smartphones for data retrieval and it was a deterrent to acceptance. Furthermore, the noninteractive nature of the Mi Band display demotivated participants from exploring the device functionalities and caused frustration with the device, which is evident from the following quote:

The Mi Band, obviously it didn’t offer as many options. And it didn’t encourage me to do as much exploring, maybe there just wasn’t—it wasn’t there. I don’t know, I kind of gave up because I couldn’t figure it out.

Anita

A majority of participants preferred Mi Band’s longer battery life of 30 days and expressed that the short battery life of the Microsoft Band negatively impacted their use:

I was able to wear it [the Microsoft Band] 2, 3 days and charge it and, you know, like you didn’t feel you had to do this all the time or you had to be home…because of my lifestyle I am not usually home at certain times.

Francine

Participants described the Microsoft Band, which is larger and inflexible, as “uncomfortable” and “rigid.” The Mi Band’s strap was preferred for its thinness and flexibility. The comfort affected participants’ decision to acceptance and use, as shown below:

Well, I have very small wrists. So, if it doesn’t fit nicely, then it’s uncomfortable and is an irritation because it’s flying around slipping down onto my hand. It’s not comfortable. So yes, it has to be comfortable to wear.

Paula

All female participants valued the aesthetics of wearable activity tracker highly in determining inappropriate settings to have them on. On the other hand, the only male participant had an indifferent opinion on the aesthetics of the device, as shown below:

Well. I wouldn’t wear them out for the evening…Not if I was going out—depending on where I’m going, but they’re definitely not formal wear.

Anita

If it looked more like jewelry, I think more people would wear it.

Paula

No [aesthetics don’t matter]. I don’t see it as a fashion thing.

Greg

Equipment characteristics were important factors for participants and they were closely tied to the perceived ease of use and perceived usefulness.

## Discussion

### Principal Findings

The aim of this study was to explore the attitudes toward and acceptance of 2 specific wearable activity trackers among a sample of Canadian community-dwelling older adults with a mixed-methods study design. A total of 20 older adults were recruited for phase 1 and 4 for phase 2. Most of the participants were frequent computer and smartphone users and had high awareness of wearable activity trackers.

Overall, participants indicated a significantly higher acceptance rate for the Mi Band (16/20, 80%) in comparison with the Microsoft Band (50%). This is also reflected in the statistical analysis where the Mi Band recorded a significantly higher score in the equipment characteristics dimension. This result emphasizes the significance of equipment characteristics in determining the acceptance for wearable activity trackers, especially as the battery life and comfort when compared between the 2 devices were substantially different. Wearable activity trackers are often regarded more personal than other technologies such as computers. This notion was well illustrated in the semistructured interview in which all female participants highly regarded wearable activity trackers as a fashion item, and the ability to conceal it was important in future decisions to use. Mercer and colleagues [30] also reported the aesthetics and subtlety of a device as major reasons for device preference.

A higher rate of acceptance for the Mi Band over the Microsoft Band was in conflict with the findings from the semistructured interviews in which the participants expressed difficulties with accessing data with the Mi Band. Mercer and colleagues [30] also reported a similar finding where older adults who were not familiar with smartphones preferred a wearable activity tracker with a clear display rather than accessing information via smartphones. Additionally, the final acceptance was higher for the device with greater comfort and style rather than its ability to display information. This may indicate that comfort, usability, battery life, and price are more crucial in accepting a wearable activity tracker than the ease of accessibility of data.

These findings have important implications for initiatives aimed at aging-in-place and the selection of technologies. To ensure long-term and continuous usage of wearable activity trackers selected to enable aging-in-place, researchers should take the steps to ensure equipment characteristics such as aesthetics, comfort, and battery life are in line with older adults’ expectations, as they have the potential to deter usage and acceptance. In this study, despite the preference of the Microsoft Band for easy data accessibility, participants reported high self-awareness of their physical activity level and health status for both devices. This warrants future research studies to investigate how varying ease of data access impacts self-awareness in finer granularity and ultimately its contribution to behavior changes.

In this study, price or how much a participant was willing to pay for a wearable activity tracker demonstrated the highest correlation (rho=.49, *P*=.001) with acceptance for both the Mi Band and the Microsoft Band. This was further confirmed through the semistructured interviews in which participants supported a greater likelihood of using a wearable activity tracker if it were free. In a systematic review that assessed technology for aging-in-place, high cost negatively influenced preimplementation technology acceptance [17]. Another study that examined the appropriateness of price in relation to the quality of product (ie, price reasonability) also identified a significant influence on technology acceptance for fitness-oriented wearable activity trackers for the general population [40].

This study is one of the first studies to confirm the significance of device cost post implementation for older adults. Understanding the relationship between the price and postimplementation acceptance is important as it indicates that the cognitive trade-off between perceived value of wearable activity trackers and monetary cost are not likely to occur for older adults. This result provides an important consideration for health promotion efforts (such as increasing physical activity, going outdoors, or increasing awareness of one’s own health) aimed toward older adults who use wearable activity trackers. Older adults who have purchased wearable activity trackers out of pocket are likely to have accepted the technology, and thus, promotion efforts should consider allocating the budget on delivering value-enhancing contents. On the other hand, health efforts that target older adults who have not considered smart wearable devices should consider subsidizing or providing the devices free of cost to increase acceptance.

Although participants claimed in the semistructured interviews that privacy was not a determining factor for using the wearable activity trackers, the quantitative results indicate the opposite. Out of the 3 privacy questions, 2 questions (L19 and L20) demonstrated a correlation between perceived privacy risk and technology rejection (rho=−.43 and −.38, respectively; *P*<.05). In other words, participants with high perceived privacy concern were moderately associated with more technology acceptance. This is in contrast with the previous research study by Gao and colleagues [40] in which perceived privacy concerns were significantly associated with wearable technology acceptance among the general population. Such inconclusive and contradicting findings may indicate that older adults had a lack of understanding of potential privacy implications. Uncertainties around privacy implications for emerging technologies were frequently identified themes among older adults [49,50]. Despite the uncertainties over privacy implications, older adults are willing to share information and compromise privacy when the technology is perceived as beneficial [17], improve or maintain independence [51], and valuable [52]. On the basis of these studies, one plausible explanation for the findings of this study is that the increased perceived benefits of wearable activity trackers may have outweighed the high privacy concerns. This may have led to the association between high technology acceptance and high privacy concern. Furthermore, Gao and colleagues [40] have also reported a weaker relationship between privacy and acceptance for medical devices compared with fitness-oriented devices. This trend indicates that older adults are willing to share fitness data when the perceived value is high. Notwithstanding, little is known about the potential privacy threats related to wearable activity trackers among older adults. In a study that investigated older adults’ perception around privacy for wearable device, a strong relationship was found between reduced perceived privacy risk and heightened and transparent legal consequences for companies [50]. The same study also discovered data sharing control as an important aspect for ensuring privacy [50]. Collection of wearable activity trackers also poses questions to public health with regard to data ownership, classification of the data as health information, and anonymity and reidentification [53]. The topic of data privacy of smart wearables and ubiquitous health will require a major attention as, currently, they are not regulated and solely rely on self-governing and oversight.

### Limitations

Several limitations are present in this study. A small sample of 20 older adults was recruited using a convenience sampling method, and the participants in this study were selected from a small number of geographical locations. This study investigated only 2 wearable activity trackers, and they may not be representative of the full range of devices and functionalities currently available. Smart wearables evolve at a very rapid rate, and the functionalities can change enormously with the introduction of new sensor technologies. While the study period was lengthier than most previous research studies of wrist-worn activity tracker acceptance, extended-term acceptance could not be comprehensively explored over a period of 6 weeks. Another limitation of this study is that it did not investigate any aspect of the data collected from the wearable activity trackers, as the purpose of this study was to understand the technology acceptance for older adults. Only 4 semistructured interviews with purposive participant recruitment were conducted. The aim of the interviews was to provide depth and context to the data collected by the questionnaires, and as such, saturation was not an end-goal. Finally, no participants of the semistructured interviews had accepted the Microsoft Band, which may have biased the qualitative results favorable to the Mi Bands.

### Conclusions

This exploratory study generated several important findings about older adults’ acceptance of and attitudes toward the 2 wrist-worn activity trackers. The acceptance of wearable activity trackers, by nature of how they are worn, is highly influenced by the equipment characteristics, including comfort, battery life, and especially aesthetics. The cost of the device is a strong indicator of technology acceptance, and its importance continues post implementation as well. Older adults were open to sharing health information generated by wearable activity trackers as long as their strengths related to health benefits and maintaining independence are clearly demonstrated. However, a lack of understanding of potential privacy risks was evident, hindering informed decision making by older adults. These findings provide guidance for future health promotion efforts that plan to target wearable activity tracker usage. Future research should focus on not only solidifying the value of smart wearables for older adults but also identifying its potential negative impact such as privacy risks. This study is a small but important step toward understanding the acceptance and usage of wrist-worn activity trackers in older adults. The findings will provide guidance for future large-scale studies.

## Appendix

Multimedia Appendix 1 Technology acceptance questionnaire.

Multimedia Appendix 2 Wilcoxon signed-ranked test for individual items from the technology acceptance questionnaire.

## Abbreviations

AUC: area under the curve
LCD: liquid-crystal display
LED: light-emitting diodes
TAM: technology acceptance model
UTAUT: unified theory of acceptance and use of technology
UV: ultraviolet
