# Supplementary material for: User Acceptance of Wrist-Worn Activity Trackers Among Community-Dwelling Older Adults: Mixed Method Study
Source: JMIR Mhealth Uhealth. 2017 Nov 15;5(11):e173. doi: 10.2196/mhealth.8211 (PMC5707431; doi:10.2196/mhealth.8211)
Supplement: Multimedia Appendix 1 [file mhealth_v5i11e173_app1.pdf]

Multimedia Appendix A

Technology Acceptance Questionnaire

<u>Item #</u>	<u>Corresponding dimension</u>	<u>Statement</u>
		1 = Strongly Disagree, 2 = Disagree, 3 = Neutral, 4 = Agree, 5 = Disagree
L1	PU	I think that monitoring my activity and health 24 hours a day, 7 days a week, can be a good thing
L2	PR	I was afraid that the device would discover a major health issue
L3	PC	I am comfortable with my health data being stored on the internet
L4	FC	I am able to get assistance from a friend of family member to use the device, if needed
L5	PEOU	I was able to wear the device easily without help from another person
L6	PEOU	I was able to remove the device easily without help from another person
L7	EC	I was able to perform my daily tasks as usual while wearing the device
L8	EC	The device was easily concealed underneath my clothing when worn
L9	EC	At times, I forgot I was wearing the device
L10	PR	I experienced skin irritations while wearing the device
L11	EC	The battery life of the device meets my expectations
L12	EC	The device's smartphone application was easy to use
L13	PEOU	I find the device easy to use
L14	PEOU	I find the display of the device easy to read indoors
L15	PEOU	I find the display of the device easy to read outdoors
L16	EC	The device was pleasant to wear during the night
L17	EC	I was concerned that the device is not securely attached to me
L18	PEOU	I was able to put the device on in a reasonable amount of time
L19	PC	I had no concerns about my privacy while wearing the device
L20	PC	I am comfortable with my health data being shared with equipment manufacturers as long as it is shared anonymously
L21	FC	I have the knowledge necessary to use the device
L22	PU	I think using the device is a more efficient way to monitor my health than visiting my doctor to collect similar information
L23	PU	Wearing the device motivated me to be more active
L24	PU	I think using the device can help me improve my overall health
L25	PR	Wearing the device caused me to have joint pain
L26	PEOU	I was able to shower or bathe normally while wearing the device

L27	SN	I was embarrassed to wear the device in front of family members
L28	SN	My friends would encourage me to use this device
L29	SN	My family members would encourage me to use this device
L30	PU	I think using the device can let me live at home longer by monitoring my health around the clock
L31	EC	The ability to use the device in a variety of locations is important to me

Additional 6 multiple-choice questions

Item #	Corresponding dimension	Statement	Answers
L32	NA	How useful did you find the information provided by the smart wearable device (such as step count, sleep data, heart rate) either on the wearable itself, or in the smartphone application?	a = very useful, b = somewhat useful, c = not very useful, d = not at all useful
L33	NA	Would you use the device you used during the last 21 days to continue to monitor or track your physical activity or health?	1 = yes, 2 = no
L34	NA	Over the last 21 days, how often do you think you wore the smart wearable device?	a = never, b = between 0-7 days, between 11-14 days, between 14-21 days
L35	NA	How much would you be willing to pay for the device you wore during the last 21 days?	a = $0, b = $1-$50, c = $51-$100, d = $101-$200, e = $201-300, f = $300-$400
L36	NA	Did you find yourself looking at your health data in the smartphone application more/less often after the first few days?	a = No, I looked at the health data fairly consistently throughout the 21-day period<br>b = Yes, I looked at the health data more often after the first few days of use<br>c = Yes, I looked at the health data less often after the first few days of use<br>d = I did not look at my health or am not interested in monitoring it
L37	NA	Do you consider yourself to be an active person?	1 = yes, 0 = no

PU: perceived usefulness

PEOU: perceived ease of use
EC: equipment characteristics
PC: privacy concern
PR: perceived risk
FC: facilitating conditions
SN: subjective norm
NA: not applicable
