# Supplementary material for: User Acceptance of Wrist-Worn Activity Trackers Among Community-Dwelling Older Adults: Mixed Method Study
Source: JMIR Mhealth Uhealth. 2017 Nov 15;5(11):e173. doi: 10.2196/mhealth.8211 (PMC5707431; doi:10.2196/mhealth.8211)
Supplement: Multimedia Appendix 2 [file mhealth_v5i11e173_app2.pdf]

Multimedia Appendix B

Wilcoxon signed ranked test for individual items in the Technology Acceptance Questionnaire

**Item #**	**Corresponding dimension**	**Mi Band Mean score (std)**	**MS Band Mean Score (std)**	**p value**
L11	H7 EC	4.55 (0.60)	2.40 (1.19)	0.0004
L16	H7 EC	3.95 (0.76)	2.60 (1.10)	0.0008
L14	H2 PEOU	3.72 (0.83)	4.40 (0.60)	0.003
L8	H7 EC	3.85 (0.88)	2.80 (1.06)	0.004
L9	H7 EC	4.20 (0.77)	3.25 (1.21)	0.005
L13	H2 PEOU	4.30 (0.66)	3.45 (1.00)	0.007
L12	H7 EC	4.00 (0.79)	3.10 (1.17)	0.007
